# Pathogenicity and Viral Shedding of MERS-CoV in Immunocompromised Rhesus Macaques

**DOI:** 10.3389/fimmu.2018.00205

**Published:** 2018-02-12

**Authors:** Joseph Prescott, Darryl Falzarano, Emmie de Wit, Kath Hardcastle, Friederike Feldmann, Elaine Haddock, Dana Scott, Heinz Feldmann, Vincent Jacobus Munster

**Affiliations:** ^1^Laboratory of Virology, Division of Intramural Research, Rocky Mountain Laboratories, National Institute of Allergy and Infectious Diseases, National Institutes of Health, Hamilton, MT, United States; ^2^Rocky Mountain Veterinary Branch, Division of Intramural Research, Rocky Mountain Laboratories, National Institute of Allergy and Infectious Diseases, National Institutes of Health, Hamilton, MT, United States

**Keywords:** Middle East respiratory syndrome coronavirus, immunosuppression, pathology, shedding, macaque monkey

## Abstract

Middle East respiratory syndrome coronavirus (MERS-CoV) has recently emerged in the Middle East. Since 2012, there have been approximately 2,100 confirmed cases, with a 35% case fatality rate. Disease severity has been linked to patient health status, as people with chronic diseases or an immunocompromised status fare worse, although the mechanisms of disease have yet to be elucidated. We used the rhesus macaque model of mild MERS to investigate whether the immune response plays a role in the pathogenicity in relation to MERS-CoV shedding. Immunosuppressed macaques were inoculated with MERS-CoV and sampled daily for 6 days to assess their immune statues and to measure viral shedding and replication. Immunosuppressed macaques supported significantly higher levels of MERS-CoV replication in respiratory tissues and shed more virus, and virus disseminated to tissues outside of the respiratory tract, whereas viral RNA was confined to respiratory tissues in non-immunosuppressed animals. Despite increased viral replication, pathology in the lungs was significantly lower in immunosuppressed animals. The observation that the virus was less pathogenic in these animals suggests that disease has an immunopathogenic component and shows that inflammatory responses elicited by the virus contribute to disease.

## Introduction

A novel coronavirus (CoV) emerged in Saudi Arabia in June of 2012 that is the causative agent of a severe respiratory disease called Middle East respiratory syndrome (MERS) (1). Thus far, there have been almost 2,100 diagnosed cases (2). Despite increased surveillance and the identification of many new cases, the case fatality rate has remained high and is currently approximately 35%.

Although there have been a high number of cases, little is known about the mechanisms of pathogenesis and the disease progression in humans is poorly described. Clinical features range from asymptomatic infection, to an acute respiratory distress syndrome, and multi-organ failure (3). The majority of patients that have succumbed to MERS-CoV have had comorbidities (4, 5) and disease is thought to be more severe in immunocompromised patients. However, the actual mechanisms of disease remain to be elucidated. The virus has been shown to replicate in human primary epithelial and *ex vivo* human lung cultures, especially in non-ciliated bronchial epithelial cells and alveolar type II pneumocytes (6–8) and the receptor has been identified as dipeptidyl peptidase 4, which is expressed on these cell types (9). MERS-CoV shedding is higher in patients with more severe disease manifestations compared to milder cases (10).

Our laboratory has recently developed two non-human primate models of MERS, utilizing the rhesus macaque and the common marmoset (11–13). Rhesus macaques develop a mild pneumonia upon intratracheal inoculation with MERS-CoV (12). In this model, virus replicates within the respiratory tract to modest levels, and is detectible in oral and nasal swabs. However, clinical disease is most prominent within the first few days after inoculation and animals show signs of disease resolution soon after. Disease in rhesus likely models the mild form of the human disease, where the infection is self-limiting and clinical signs and symptoms are mild (10, 14, 15). In an effort to examine whether the immune status of an individual influences the disease severity and pathogenicity and replication kinetics of the virus, we downregulated the immune system of rhesus macaques using immunosuppressive drugs. We found that MERS-CoV replicated to significantly higher titers and disseminated outside of the respiratory tract in immunosuppressed animals, yet pathology was markedly reduced in these animals, showing that disease has an immunopathogenic component.

## Materials and Methods

### Ethics Statement

The use of study animals was approved by the Institutional Animal Care and Use Committee of the Rocky Mountain Laboratories and experiments were performed following the guidelines of the Association for Assessment and Accreditation of the Laboratory Animal Care by certified staff in an approved facility. The guidelines and basic principles in the United States Public Health Service Policy on Humane Care and Use of Laboratory Animals and the Guide for the Care and Use of Laboratory Animals were followed. All procedures were carried out under anesthesia using Ketamine by trained personnel under veterinarian supervision and efforts were made to provide for the welfare of animals and to minimize suffering. All animals were humanely euthanized at the endpoint of the study (6 days post-inoculation) by exsanguination under deep anesthesia. All standard operating procedures for MERS-CoV were approved by the Institutional Biosafety committee of the Rock Mountain Laboratories, and sample inactivation was carried out according to approved standard operating procedures prior to removal from high containment.

### Virus Propagation

Middle East respiratory syndrome coronavirus (isolate EMC/2012) was propagated in Vero E6 cells in DMEM (Sigma) supplemented with 2% FBS (Logan), 1 mM l-glutamine (Lonza), 50 U/mL penicillin, and 50 μg/mL streptomycin (both from Gibco).

### Rhesus Macaque Immunosuppression and Inoculation

Five Rhesus macaques (female, weighing 7–11 kg, 11 years of age) were enrolled in this study. Immunosuppression (animals ISCoV1-3) was achieved by administration of cyclophosphamide (CyP) (Roxane Laboratories) (10 mg/kg dissolved in 30 mL of a meal supplement (Boost) and delivered *via* an orogastric tube under anesthesia every other day starting 16 days prior to virus inoculation and ending 2 days after inoculation), and dexamethasone (Dex, 2 mg/kg daily by subcutaneous injection beginning 16 days prior to virus inoculation and ending 5 days after inoculation). Mock immunosuppression (CoV1-2) was performed following the same schedule, but orogastric feeding did not contain CyP and injections consisted of sterile PBS. Immunosuppression was confirmed by monitoring white blood cell (WBC) populations using a HemaVet (Drew Scientific). For inoculation, 7 × 10^6^ TCID_50_ of MERS-CoV was diluted in 7 mL of DMEM and delivered *via* intratracheal (4 mL) oral (1 mL) nasal (1 mL), and ocular (1 mL) routes as previously described (12). Clinical exams were performed on days −18, −16, −10, −4, −2, 0, and +1 to +6 relative to virus inoculation. Blood was obtained at these times as well as nasal and oral swabbing and chest radiographs starting on day 0. Six days after inoculation, all five animals were euthanized and necropsies performed to obtain samples of the following tissues: lungs (all six lobes), bronchi, oro/nasopharynx, trachea, tonsils, heart, liver, spleen, kidney, adrenal gland, pancreas, inguinal, axillary, mesenteric, and mediastinal lymph nodes.

### Virus Quantitation

We used a one-step real-time quantitative RT-PCR to measure viral RNA in the samples. RNA was extracted from swabs using the QiaAmp Viral RNA extraction kit and tissues using the RNeasy kit (both from Qiagen). RNA was then used along with a MERS-CoV-specific primer/probe set using the Rotor-Gene Probe kit (Qiagen). Tissue culture infectious dose 50% (TCID50) equivalents were calculated by comparing cycle threshold values to a standard curve generated from virus stocks of known titer. Primers and probe sequences were described previously (16).

### Histopathology

Tissues were fixed in 10% neutral buffered formalin with two changes, for a minimum of 7 days and processed with a Sakura VIP-5 Tissue Tek, on a 12 h automated schedule, using a graded series of ethanol, xylene, and ParaPlast Extra. Embedded tissues were then sectioned at 5 μm and dried overnight at 42°C prior to staining.

For immunohistochemistry (IHC), tissues were processed using the Discovery XT automated processor with a DAPMap kit (both from Ventana). Specific primary antibodies used were: anti-HCoV-EMC polyclonal rabbit antibody (17) against CoV at a 1:1,000 dilution, anti-CD3 (2GV6) rabbit monoclonal primary antibody applied neat, and anti-CD20 (Thermo Scientific) at a 1:100 dilution. For CD3 and CD20, IHC stained sections were scanned with an Aperio ScanScope XT (Aperio Technologies, Inc., Vista, CA, USA) and analyzed using the ImageScope Positive Pixel Count algorithm (version 9.1). Approximately 25 mm squared were evaluated at 2× magnification. The default parameters of the Positive Pixel Count (hue of 0.1 and width of 0.5) were used.

## Results

### Rhesus Macaque Immunosuppression

**Figure 1:**
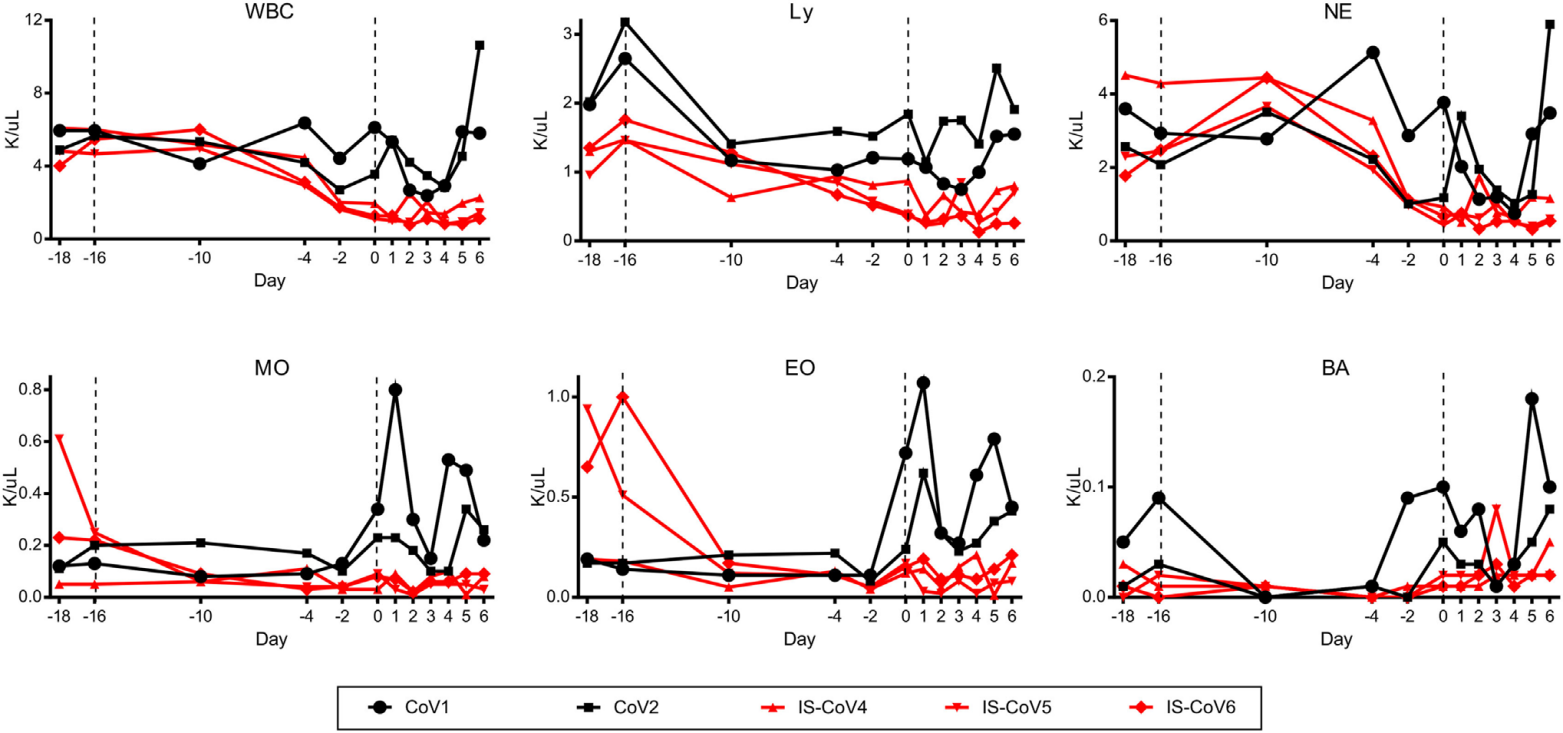
Immunosuppression of rhesus macaques. Three rhesus macaques were immunosuppressed using a combination of cyclophosphamide (10 mg/kg every other day) and dexamethasone (2 mg/kg daily) for 16 days (−16) prior to inoculation with Middle East respiratory syndrome coronavirus, and for 5 days thereafter. Blood samples were collected at the indicated time points, inclusive of a control bleed 2 days prior to the initiation of the study (−18), and blood cells were enumerated. All blood cell types measured were decreased in the immunosuppressed animals (red lines) at the time of inoculation and remained depressed. Control animals given PBS (black lines) had no reduction in cell counts and cell counts increased in response to infection. Total white blood cells (WBC), lymphocytes (Ly), neutrophils (NE), monocytes (MO), eosinophils (EO), and basophils (BA) were measured.

**Figure 2:**
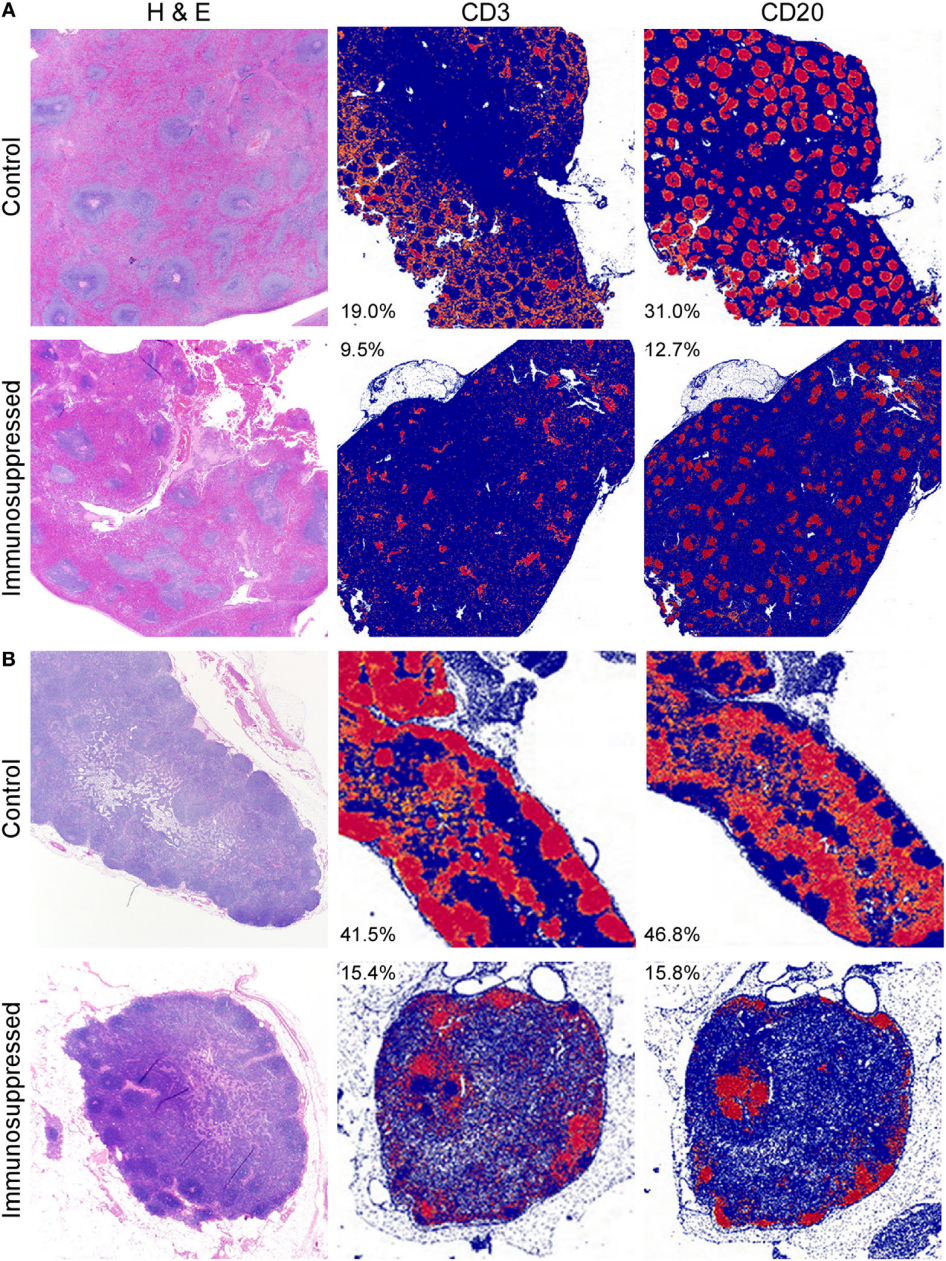
Depletion of immune cells in lymphoid tissues. Tissues collected from control and immunosuppressed animals at the time of euthanasia (6 dpi) were stained with H&E or markers for T cells (CD3) and B cells (CD20). The spleens **(A)** and mediastinal lymph nodes **(B)** of immunosuppressed animals showed disruption of the normal architecture of the white pulp visualized after H&E staining. Quantitation of the amount of specific CD marker staining (red), compared to the absence of staining (blue) showed that the amount of CD3 staining was reduced approximately 2-fold in the spleen **(A)** and 2.7-fold in the lymph node **(B)** in the immunosuppressed animals. Likewise, CD20 staining was reduced by 2.4-fold in the spleen and 3-fold in the lymph nodes, showing a reduction in these cells. Values shown are the percent of positive (red) staining within a tissue and are the average for all animals within each group.

To assess the contribution of the immune response to protection from MERS disease, we immunosuppressed three rhesus macaques using CyP and Dex for 16 days prior to inoculation with 7 × 10^6^ TCID_50_ of MERS-CoV. Two animals were used for mock immunosuppression controls and received the identical inoculum. Throughout immunosuppression, we monitored WBC populations in the blood to determine the efficacy of the immunosuppression regimen. Total WBC counts were decreased by approximately twofold compared to the control animals in response to CyP and Dex administration at the time of MERS-CoV inoculation. This reduction was due to decreases in all measured cell types (lymphocytes, neutrophils, monocytes, eosinophils, and basophils) (Figure 1). Following MERS-CoV inoculation, the absolute counts of these cell populations remained low in the immunosuppressed animals, suggesting their inability to respond to infection. Conversely, the two mock immunosuppressed animals had increased numbers of monocytes and eosinophils in response to infection. To quantify immunosuppression at the tissue level, the spleens and mediastinal lymph nodes of all animals were stained immunohistochemically with T cell and B cell markers (CD3 and CD20, respectively) post-mortem. The amount of staining was quantitatively assessed using imaging software. The quantity of CD3 staining was approximately 2-fold lower in the spleen and 2.7-fold in a mediastinal lymph node in the immunosuppressed animals. Likewise, CD20 was 2.4-fold lower in the spleen and 3-fold lower in the lymph nodes, showing a general reduction in lymphocytes in these tissues and confirming that the suppressive drug therapy was effective in reducing immune cell populations (Figures 2A,B). Immunosuppression also disrupted the normal architecture of these tissues.

### Virus Shedding and Replication

**Figure 3:**
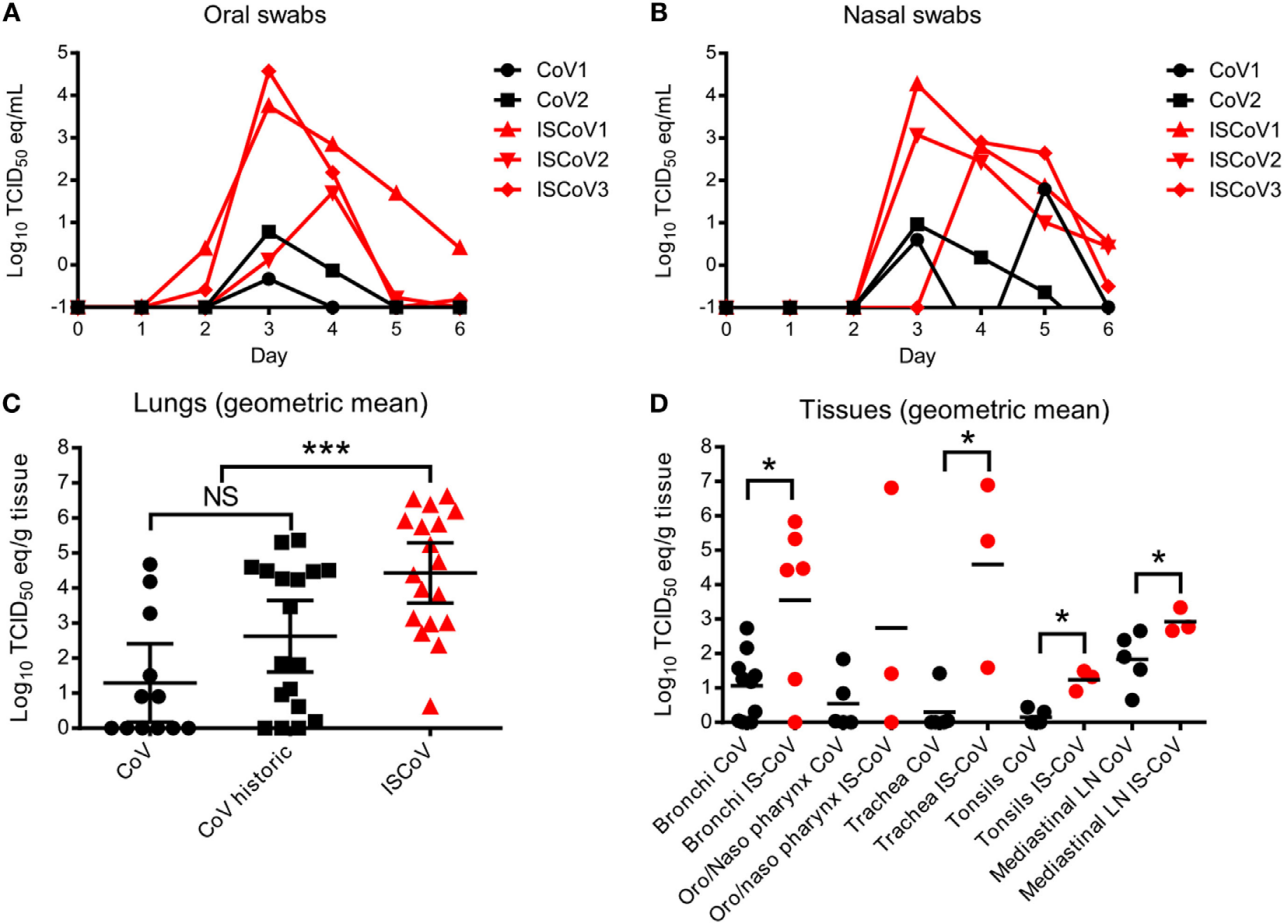
Middle East respiratory syndrome coronavirus shedding and replication. Shedding of virus was monitored by measuring viral RNA in nasal **(A)** and oral **(B)** swabs using MERS-CoV-specific qRT-PCR. Nasal swabs were positive for viral RNA for all but one immunosuppressed animal starting 3 dpi, with immunosuppressed animals (red lines) reaching higher titers and more sustained shedding. Oral swabs **(B)** showed a similar trend. Viral replication was assessed in the lungs **(C)** by performing qRT-PCR using tissue samples from each of the six lobes from each animal. Data are represented as the geometric mean and 95% confidence interval from each group. qRT-PCR was performed on an additional three animals from a previous macaque study (CoV historic) to compare to the immunosuppressed animals. Tissues from the respiratory tracts were also analyzed for RNA levels **(D)** and the samples from the historic control macaques were grouped with the two control animals from this study (black circles). Data are represented as the geometric means of the groups. To compare the data sets within **(C,D)**, a Kolmogorov–Smirnov test was performed to determine statistical differences between the control and immunosuppressed groups. Asterisks (*) indicate significant differences, with *p ≤ 0.05 and ***p ≤ 0.001.

Oral and nasal swab samples were obtained daily throughout the course of MERS-CoV infection to monitor the shedding of viral RNA. While shedding was detected from all animals, detection of viral RNA occurred earlier, persisted longer, and was several logs higher, in the immunosuppressed animals compared to the control animals (Figures 3A,B). Viral RNA was detectable at 6 days post-inoculation in nasal swabs from all three immunosuppressed animals and oral swabs from two animals, whereas the control animals were negative by this time point.

To assess virus replication in the tissues, we enlisted three rhesus macaques from a previous study to serve as historic controls, along with the two controls in this study (11). All animals were given the same inoculum (from the same stock of virus) *via* the same route and all were euthanized 6 days post-inoculation. When comparing the viral abundance in the lungs (all six lobes) between the controls from this study and the historic controls, there was no significant difference in the geometric means of viral RNA abundance between these two groups, although the historic controls had slightly more measurable viral RNA (Figure 3C). However, the immunosuppressed animals had significantly increased MERS-CoV replication (measured by RNA abundance) in the lungs (Kolmogorov–Smirnov test, *p* ≤ 0.001). Similarly, there was significantly more virus detected in several respiratory, or respiratory tract-associated tissues, including the bronchi, trachea, tonsils, and mediastinal lymph nodes of immunosuppressed animals, compared to the controls (Figure 3D) (Kolmogorov–Smirnov test, *p* ≤ 0.05). When assessing viral dissemination in tissues outside of the respiratory tract, immunosuppressed animals were positive for low levels of viral RNA in several tissues, including the liver and spleen, as well as several lymph nodes, whereas virus was undetectable outside of the respiratory tract in the control animals, with the exception of one inguinal lymph node sample (Table 1).

**Table 1:** Middle East respiratory syndrome coronavirus (MERS-CoV) dissemination in rhesus macaque tissues detected by qRT-PCR.

Tissue	CoV1	CoV2	IS-CoV1	IS-CoV2	IS-CoV3
Heart	–	–	–	+	–
Liver	–	–	+	–	+
Spleen	–	–	+	–	+
Kidney	–	–	–	–	+
Adrenal gland	–	–	+	–	–
Pancreas	–	–	+	+	–
Inguinal LN	–	+	+	+	+
Axillary LN	–	–	+	+	+
Mesenteric LN	–	–	+	+	+

*(–) indicates that no viral RNA was detected, (+) indicates that viral RNA was detected by qRT-PCR. LN, lymph node*.

### Lung Histopathology

**Figure 4:**
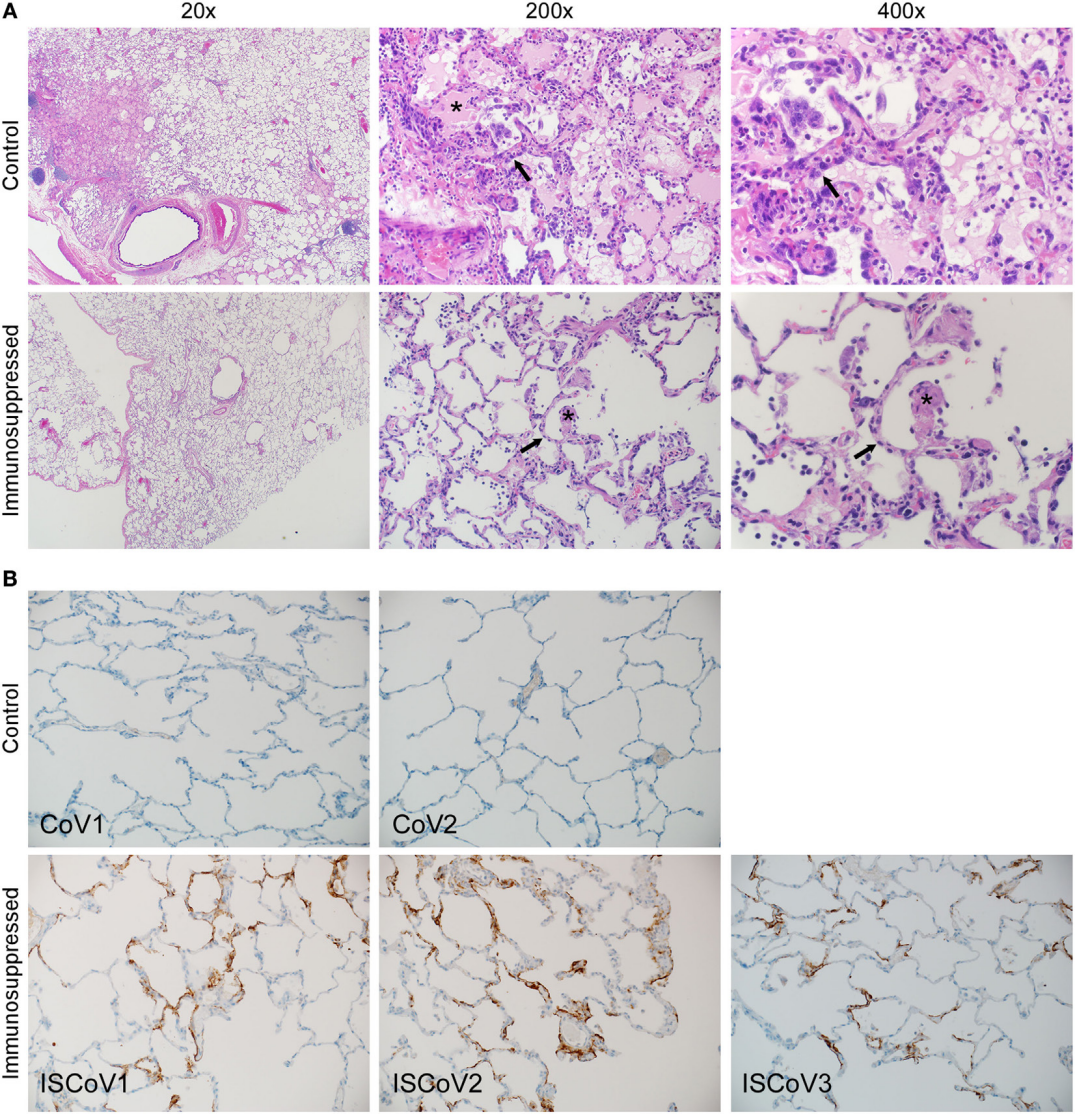
Histopathologic changes induced by Middle East respiratory syndrome coronavirus (MERS-CoV). Lung tissue samples from control and immunosuppressed animals were stained with H&E **(A)** or an antibody specific for MERS-CoV **(B)**. Lung tissue from representative animals from each group were stained with H&E **(A)** and demonstrate that control animals developed multifocal, mild-to-marked, interstitial pneumonia, and thickening of alveolar septae by congestion, edema and fibrin, and formation of hyaline membranes. Multifocal type II pneumocyte hyperplasia was noted. The lungs of immunosuppressed macaques showed few pathologic changes. Asterisks show fibrin deposits and arrows indicate edema. Immunohistochemistry **(B)** of control animals showed little or no viral antigen present. By contrast, immunosuppressed animals showed significant levels of viral antigen, which was multifocal throughout the tissue and predominant within type I pneumocytes.

Samples from all animals were evaluated for the presence of histopathologic changes. Each of the animals, with the exception of IS-CoV3, developed some degree of pulmonary pathology upon examination of tissue following necropsy 6 days after inoculation (Figure 4A). Lesions were characterized as multifocal, mild-to-marked, interstitial pneumonia and were frequently centered on terminal bronchioles. The pneumonia was characterized by thickening of alveolar septae by congestion, edema and fibrin, and small to moderate numbers of macrophages and neutrophils. Alveoli contained moderate numbers of pulmonary macrophages and neutrophils. In lungs with marked changes, there was abundant alveolar edema and fibrin with the formation of hyaline membranes. Multifocal type II pneumocyte hyperplasia was noted and there were also perivascular infiltrates of inflammatory cells within, and adjacent to, affected areas of the lung. Samples from each lung lobe for each animal were individually scored for the presence and extent of pathologic changes, with scores ranging from 0 (no pathology) to 4 (multiple coalescing inflammatory foci with fibrin and edema). Animals CoV1 and CoV2 had average histology scores of 1.3 and 1.2, with individual lung lobe scores ranging from 0 to 4 and from 0 to 3, respectively (Table 2). Immunosuppressed animals displayed much milder pathology with average scores for IS-CoV1, IS-CoV2, and IS-CoV3 of 0.2, 0.6, and 0, respectively, and no lobe showing a score greater than 1.

**Table 2:** Histopathologic scoring of lungs from rhesus macaques.

Tissue	CoV1	CoV2	ISCoV1	ISCoV2	ISCoV3
Right upper lobe	0	1	0	1	0
Right middle lobe	2	0	1	1	0
Right lower lobe	0	1	0	0	0
Left upper lobe	2	0	0	0	0
Left middle lobe	0	2	0	1	0
Left lower lobe	4	3	0	1	0
Average score	1.3	1.2	0.2	0.6	0

*(0) = no pathology, (1) = few inflammatory foci scattered between multiple lung lobes; alveolar interstitium minimally thickened by congestion and small numbers of neutrophils and macrophages; few neutrophils and macrophages within alveoli, (2) = multiple inflammatory foci in multiple lung lobes; alveolar interstitium is mildly thickened, edema and small numbers of neutrophils and macrophages; small numbers of neutrophils and macrophages within alveoli, (3) = multiple inflammatory foci scattered within single lung lobes; alveolar interstitium is moderately thickened, edema and moderate numbers of neutrophils and macrophages; many neutrophils and macrophages within alveoli; small amounts of fibrin and edema in alveoli, (4) = multiple to coalescing inflammatory foci within a single lung lobe; alveolar interstitium is markedly thickened by congestion, edema, fibrin, and large numbers of neutrophils and macrophages; large numbers of neutrophils, macrophages, cellular debris, fibrin, and edema within alveoli*.

The animals that did not undergo immunosuppression developed the most severe pulmonary pathology, but demonstrated little or no viral antigen in the lung tissue examined by IHC (Figure 4B), reflecting the qRT-PCR results, where much less viral RNA was detected and viral RNA was undetectable in many of the individual lung lobes. Conversely, macaques that had undergone immunosuppression had very mild lung lesions, but demonstrated MERS-CoV viral antigen multifocally throughout the lung; predominantly within type I pneumocytes. This suggests that pulmonary pathology associated with MERS-CoV in these animals may be tightly associated with the immune response.

## Discussion

Little is known regarding how several emerging zoonotic viruses infecting the respiratory tract cause disease, and what risk factors contribute to poor outcome. Some viruses are thought to cause disease by dysregulating the immune response, whereby destruction of infected cells or secretion of pro-inflammatory mediators leads to immunopathology, as in the case of hantaviruses (18). Conversely, pathogenesis caused by some viruses correlates with deficiencies, or inefficiencies of the immune response, such as in the case of pathogenic viruses affecting the very young and elderly, or immune compromised individuals, as in the case of influenza virus (19). The mechanisms by which the recently emerged MERS-CoV causes disease in humans, and what host factors are associated with either resistance or a poor outcome are not known (15). These questions are important for the development of countermeasures that either directly target the virus to inhibit its replication or modulate the immune response to limit immunopathogenesis.

Early epidemiologic studies of MERS suggested that patients with comorbidities fared worse than healthy patients upon infection, and the number of comorbidities correlated with a worse outcome (20). In addition, a few patients with lethal infections were reported to be immunosuppressed (21–23). These reports were from cases where diagnoses were primarily in patients already in hospitals, including a relatively large number of nosocomial infections affecting 23 patients in a hospital outbreak in Al-Hasa (24). Since these initial case reports, more than 2,000 additional cases of MERS-CoV infection have been confirmed (2). Although many of these new cases are reported to be health care related, either stemming from patients or health care workers, it is unclear how many of these cases involve immunocompromised individuals.

Risk factors that have been associated with disease (or infection) include weakened immune systems and chronic diseases, such as diabetes, cancer, and chronic lung disease, as well as co-infections (5, 20, 25). Although these comorbidities clearly affect the status of the immune response, acute immunosuppression using drugs, as we have done here, provides a more controlled deficit in immune responses, whereas immune dysregulation brought on by chronic disease, infection, and aging is a complex phenomenon that involves deficiencies in the immune response, chronic inflammatory responses, and other known and yet to be described complex changes. For instance, the majority of patients had diabetes as a comorbidity and several others were immunosuppressed with HIV/AIDS. Both of these conditions alter the immune response in a way that both inhibits normal T cell functions, as well as inducing an inflammatory response by altering Th17 responses and secretion of inflammatory cytokines (26–30). The immunosuppression in our study mimics some aspects of the human condition in these patients, such as inhibition of CD4^+^ T cell responses by HIV-infected patients. However, the chemical immunosuppression used herein is unlikely to mimic the chronic inflammatory state in many of these patients. This higher basal level of immune activation associated with these conditions may be important contributions to the manifestation of the clinically overt serious disease following MERS-CoV infection, and would imply that the immune system plays a role in the pathogenesis of MERS-CoV. This agrees with our observation that upon simple immunosuppression, MERS-CoV replicated to higher levels and showed greater dissemination and shedding, while the pathology was actually reduced in these animals. Pathology was likely lessened due to the absence of inflammatory cells and mediators, as observed histologically in the lung tissues. This suggests that the virus itself might cause little damage to the cells that it infects and this would lead toward a mechanism in which the absence of an efficient immune response allows the virus to replicate to high levels, whereas pathology can be attributed to the overactive inflammatory response, which patients with comorbidities are prone to possess. This is supported by data in the resus and marmoset animal models, which show that increased viral replication and the local immune response to this plays an important role in the pulmonary severity of disease (31). Although not performed in this study, a control group treated with immunosuppressive drugs, and not challenged, would be necessary for a comprehensive picture of the immune status of these animals at the time of necropsy.

Recent experiments using human-derived blood cells have shown that infection with MERS-CoV results in a dramatic increase in the production of cytokines and immune cell-recruiting chemokines and the authors hypothesize that these inflammatory responses could lead to severe inflammation and tissue damage (32, 33). This is supported by the observation that bronchoalveolar lavage fluid of humans infected with MERS-CoV contain high numbers of neutrophils and macrophages (1, 22). Furthermore, lymphopenia has been associated with disease and is potentially caused by infiltration of lymphocytes in the lung tissue and egress from the blood (34). Taking these findings into account, we can envision a model in which infection of the lung tissue and resident immune cells, such as alveolar macrophages, leads to the hyper-production of inflammatory cytokines and immune cell recruitment chemokines, which together limit virus replication, but result in an immunopathologic state. Upon immunosuppression of our macaques, the virus was still able to infect and replicate in the lung tissue, and likely induced local cytokine and chemokine expression; however, the depletion of immune cell populations upon chemical immunosuppression inhibited recruitment of inflammatory cells to the lungs (or infected tissues) and, thus, limited pathology. This is the first direct experimental evidence showing that MERS-CoV has an immunopathogenic component. This is in line with the observation in one patient in South Korea, which was taking prolonged high-dose corticosteroid therapy to control lymphoma activity and hemolytic anemia and displayed persistant viral shedding without clinical progression of the disease (23).

The shedding of MERS-CoV was more extensive in the immunosuppressed animals, both in duration as in peak shedding. This suggests that the immune status has direct influence on virus shedding and subsequent potential of transmission. The epidemiological analyses of the 2015 MERS-CoV outbreak in South Korea clearly showed that only the level of MERS-CoV shedding was directly associated with transmission potential. Where spreaders had statistically lower Ct values compared to non-spreaders (25). The persistent MERS-CoV shedding in immunocompromised patients (23) could, therefore, contribute to enhanced nosocomial transmission.

As of yet, no specific treatment options have been identified for MERS-CoV infection. The results presented herein show that inflammatory responses contribute to the pathogenic process. This would suggest that treatment for patients with symptomatic infections would benefit from additional therapy that lessens the inflammatory response, especially in the lung, and not be based solely on therapies that are aimed at controlling virus replication.

## Ethics Statement

The use of study animals was approved by the Institutional Animal Care and Use Committee of the Rocky Mountain Laboratories and experiments were performed following the guidelines of the Association for Assessment and Accreditation of the Laboratory Animal Care by certified staff in an approved facility. The guidelines and basic principles in the United States Public Health Service Policy on Humane Care and Use of Laboratory Animals and the Guide for the Care and Use of Laboratory Animals were followed. All procedures were carried out under anesthesia using Ketamine by trained personnel under veterinarian supervision and efforts were made to provide for the welfare of animals and to minimize suffering. All animals were humanely euthanized at the endpoint of the study (6 days post-inoculation) by exsanguination under deep anesthesia. All standard operating procedures for MERS-CoV were approved by the Institutional Biosafety committee of the Rock Mountain Laboratories, and sample inactivation was carried out according to approved standard operating procedures prior to removal from high containment.

## Author Contributions

JP, DF, EW, HF, and VM designed the study; JP, DS, HF, and VM analyzed the data; JP, HF, and VM wrote the manuscript; and JP, KH, FF, EH, VM, and DS performed the experiments and assays.

## Conflict of Interest Statement

The authors declare that the research was conducted in the absence of any commercial or financial relationships that could be construed as a potential conflict of interest.
